# Supplementary material for: Structural and electronic determinants of lytic polysaccharide monooxygenase reactivity on polysaccharide substrates
Source: Nat Commun. 2017 Oct 20;8:1064. doi: 10.1038/s41467-017-01247-3 (PMC5651836; doi:10.1038/s41467-017-01247-3)
Supplement: Supplementary file 1 — Supplementary Information [file 41467_2017_1247_MOESM1_ESM.pdf]

```
              1        10       20       30       40         50
LsAA9A|       HTLVWGVWVNGVDQGDGRNIYIRSPPNNNPVKNLTSPDMTCNVDN-RVVPKSVPVNAGDT
CvAA9 |       HTRMFSVWVNGVDQGDGQNVYIRTPPNTDPIKDLASPALACNVKGGEPVPQFVSASAGDK
              ** ::.***********:*:***:***.:*:*:*:** ::***.. . **: *...***.

              60        70        80        90           100       110
LsAA9A|       LTFEWYHNTRDDDIIASSHHGPIAVYIAPAAS---NGQGNVWVKLFEDAYNVTNSTWAVD
CvAA9 |       LTFEWYRVKRGDDIIDPSHSGPITTWIAAFTSPTMDGTGPVWSKIHEEGYDASTKSWAVD
              ******: .*.**** .** ***:.:**. :*   :* * ** *:.*:.*:.:..:****

                 120       130        140       150       160       170
LsAA9A|       RLITAHGQHSVVVP-HVAPGDYLFRAEIIALHEADSLYSQNPIRGAQFYISCAQITINSS
CvAA9 |       KLIANKGMWDFTLPSQLKPGKYMLRQEIVAHHESDATFDKNPKRGAQFYPSCVQVDVKGV
              :**: :*  ...:* :: **.*::* **:* **:*: :.:** ****** **.*: ::.

                  180       190       200       210       220       230
LsAA9A|       DDSTPLPAGVPFPGAYTDSTPGIQFNIYTTPATSYVAPPPSVWSGALGGS--IAQVGDAS
CvAA9 |       GGDAVPDQAFDFNKGYKYSDPGIAFDMYTD-FDSYPIPGPPVWDAQDEGCCFIDGVDTTS
              ...:    .. *  .*. * *** *::**    **  * *.**..   *.  *  *. :*

LsAA9A|       LE-----------
CvAA9 |       VKEVVKQIICVLK
              ::
```

Supplementary Figure 1: Alignment of *Ls*AA9A and *Cv*AA9A enzymes. Copper ligand residues are highlighted in yellow; key residues of *Ls*AA9A involved in protein:substrate interactions are highlighted in green and the equivalent residues of *Cv*AA9A are in purple.

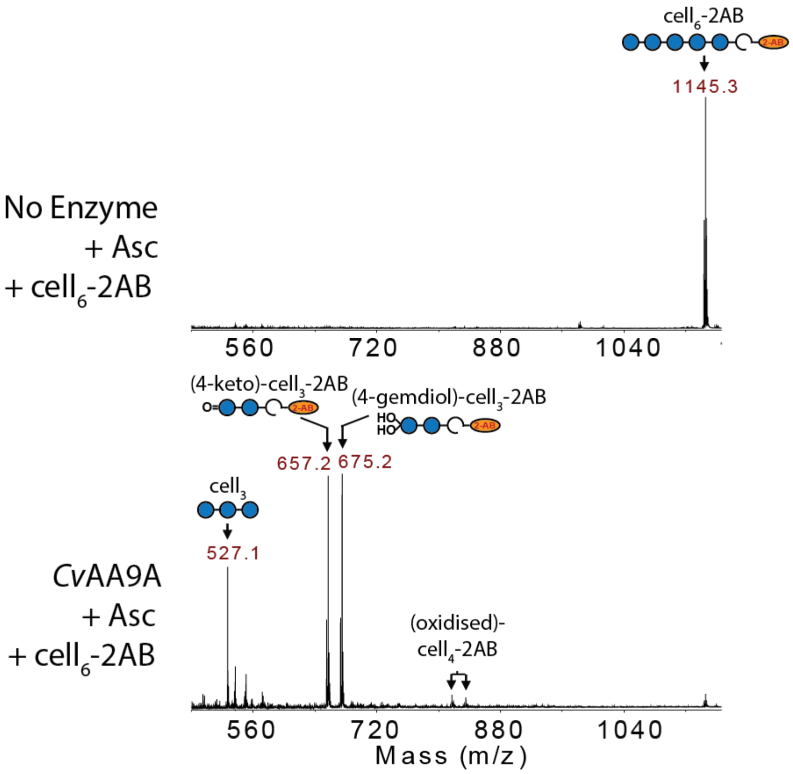

Supplementary Figure 2: *Cv*AA9A cleaves Cell$_6$-2AB using solely a C4-oxidation mechanism. MALDI-ToF MS spectra showing substrates and products of *Cv*AA9A activity on Cell$_6$-2AB.

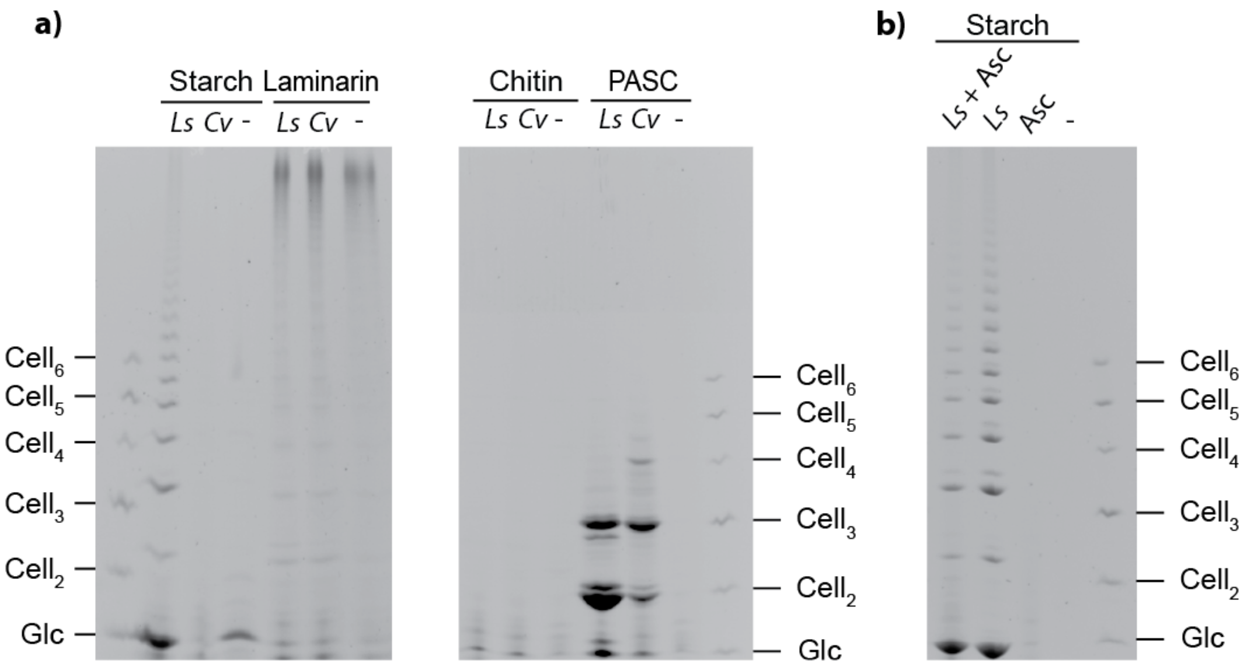

Supplementary Figure 3: *Ls*AA9A and *Cv*AA9A are unable to cleave a range of polysaccharide structures. a, PACE gels showing products of *Ls*AA9A and *Cv*AA9A activity on a range of polysaccharide substrates with 4mM ascorbate. **b,** PACE gel showing products of *Ls*AA9A on starch, showing that cleavage by the *Ls*AA9A preparation is not reductant-dependent and therefore likely the product of a contaminating hydrolase. Ls, *Ls*AA9A; Cv, *Cv*AA9A; Asc, ascorbate.

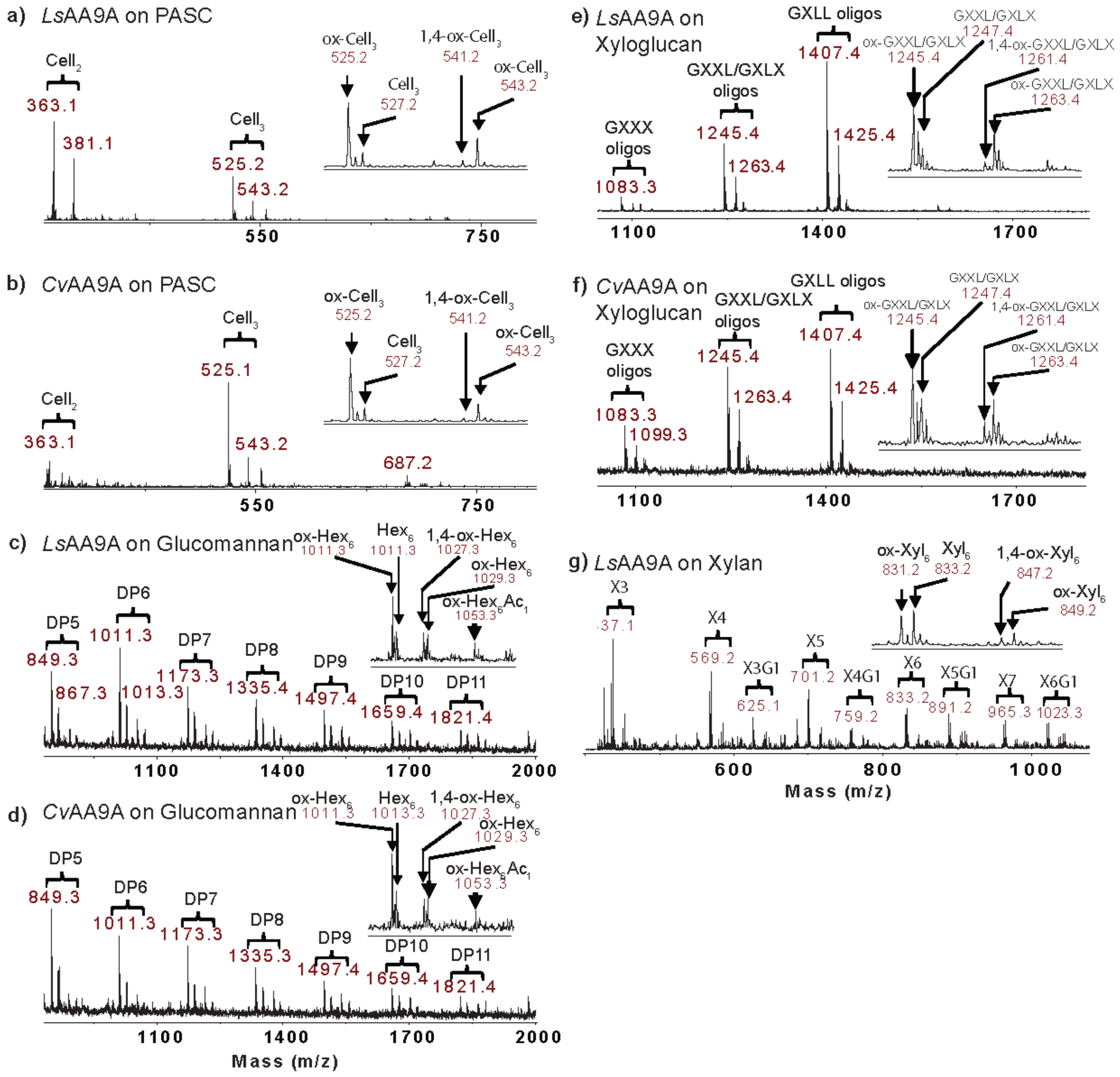

Supplementary Figure 4: MALDI ToF MS spectra of the products of *Ls*AA9A and *Cv*AA9A on a range of polysaccharide substrates. DP, degree of polymerization; ox-, oxidized oligosaccharides. For xyloglucan nomenclature, see Fry et al[1].

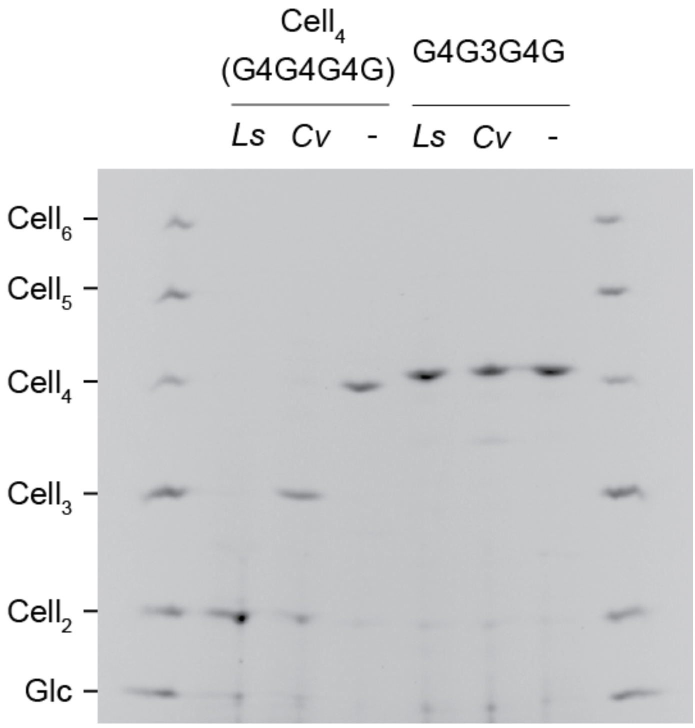

Supplementary Figure 5: *Ls*AA9A and CvAA9A are able to cleave Cell$_4$ but not G4G3G4G. PACE gel showing products of *Ls*AA9A and *Cv*AA9A activity on Cell$_4$ but not G4G3G4G (D-Glc-β-(1→4)-D-Glc-β-(1→3)-D-Glc-β-(1→4)-D-Glc), with 4mM ascorbate.

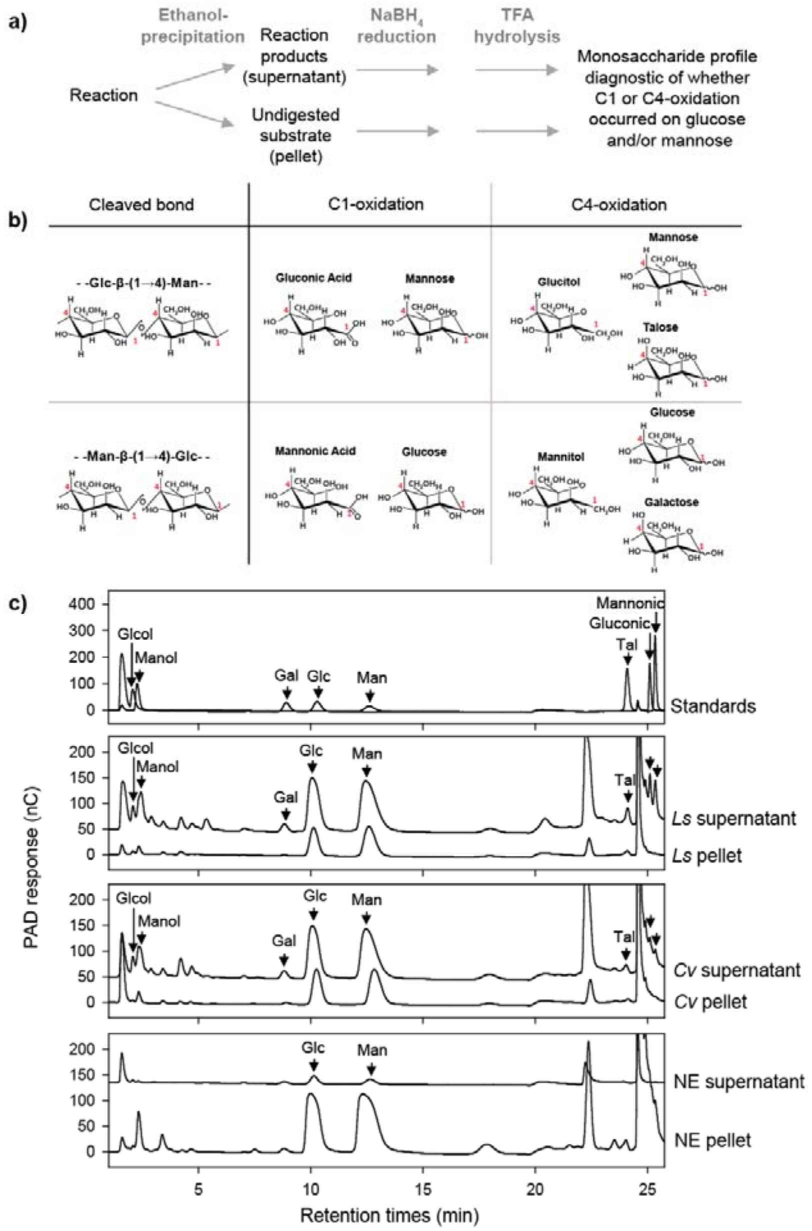

Supplementary Figure 6: *Ls*AA9A and *Cv*AA9A can cleave glucomannan with glucosyl or mannosyl residues at subsites -1 and +1 and yield both C1- and C4-oxidised products a, Protocol for analysis of site of cleavage and oxidation state **b,** Expected products (following analysis protocol) after C1 or C4 oxidative cleavage of different bonds. **c,** HPAEC analysis of products (supernatant) and undigested substrates (pellet) of *Ls*AA9A and *Cv*AA9A activity on glucomannan. The presence of Talose indicates C4-oxidation of mannose. The presence of mannonic acid indicates C1 oxidation of mannose. All reactions using 4mM ascorbate as reductant. NE, no enzyme. Tal, Talose; Glcol, glucitol; Manol, Mannitol.

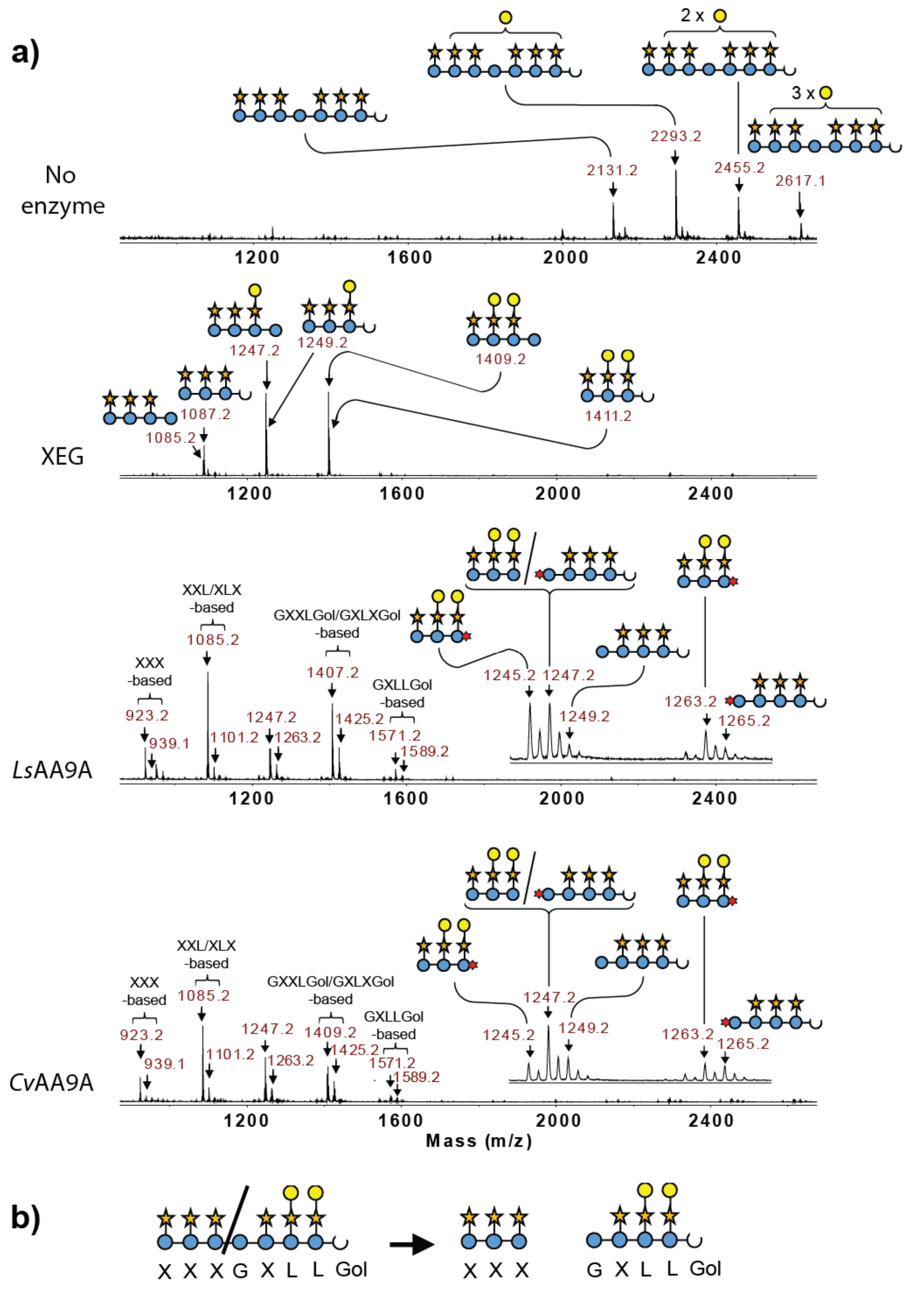

Supplementary Figure 7: *Ls*AA9A and *Cv*AA9A cleave xyloglucan oligosaccharides with unsubstituted glucose at subsite +1. a, MALDI MS spectra showing products of *Ls*AA9A and CvAA9A activity on a range of di-subunit xyloglucan oligosaccharides. The LPMOs cleave at a different site to xyloglucan endoglucanase (XEG). **b,** Schematic diagram showing cleavage of a xyloglucan oligosaccharide.

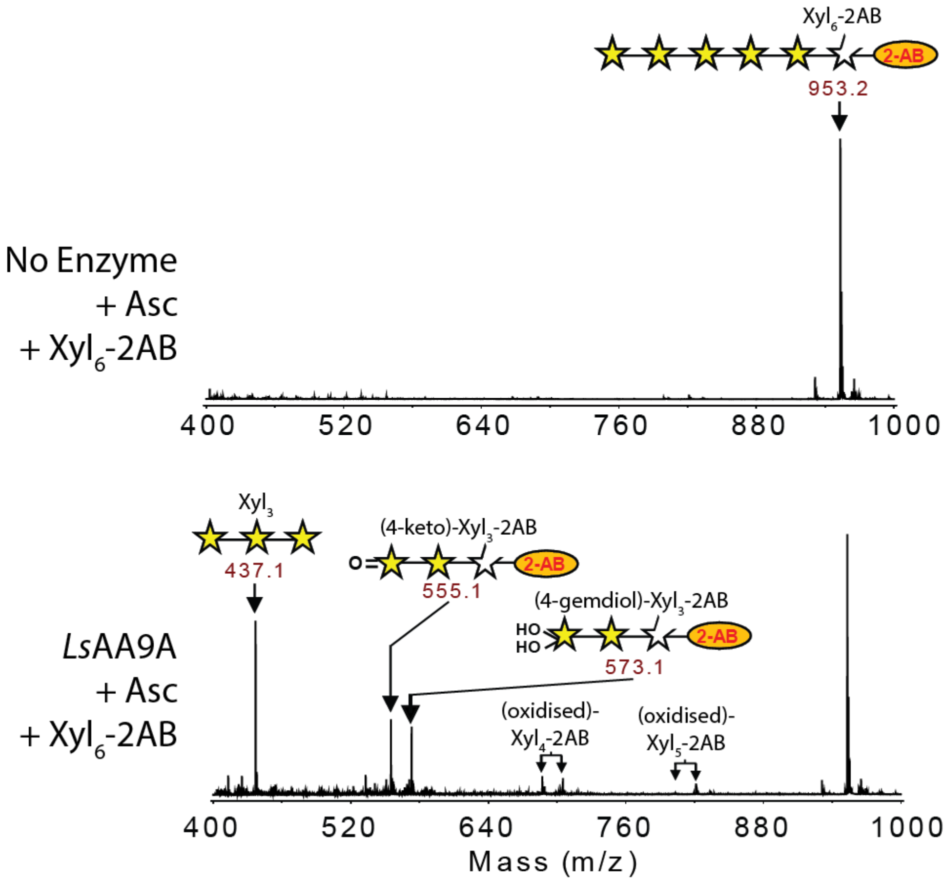

Supplementary Figure 8: *Ls*AA9A cleaves Xyl$_6$-2AB using solely a C4-oxidation mechanism. MALDI MS spectra showing substrates and products of *Ls*AA9A activity on Xyl$_6$-2AB.

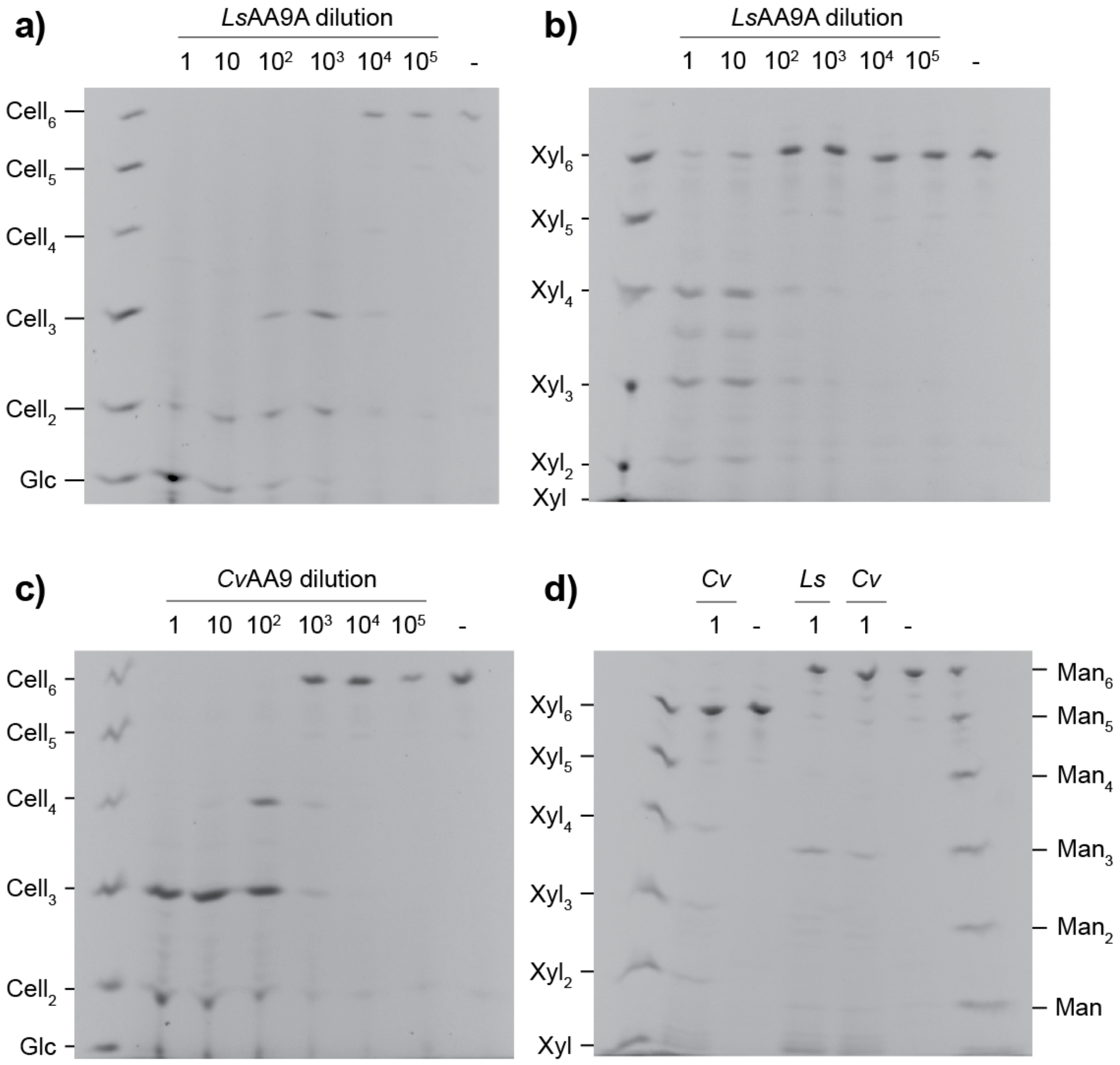

Supplementary Figure 9: *Ls*AA9A cleavage of Xyl_6 is much faster than *Cv*AA9A. PACE gels showing cleavage of oligosaccharides by different dilutions from standard assay conditions of *Ls*AA9A and *Cv*AA9A with 4mM ascorbate. a *Ls*AA9A on $Cell_6$, b *Ls*AA9A on Xyl_6 c CvAA9A on $Cell_6$ d CvAA9A shows no activity of Xyl_6. *Ls*AA9A and *Cv*AA9A show scarcely detectable activity on Man_6

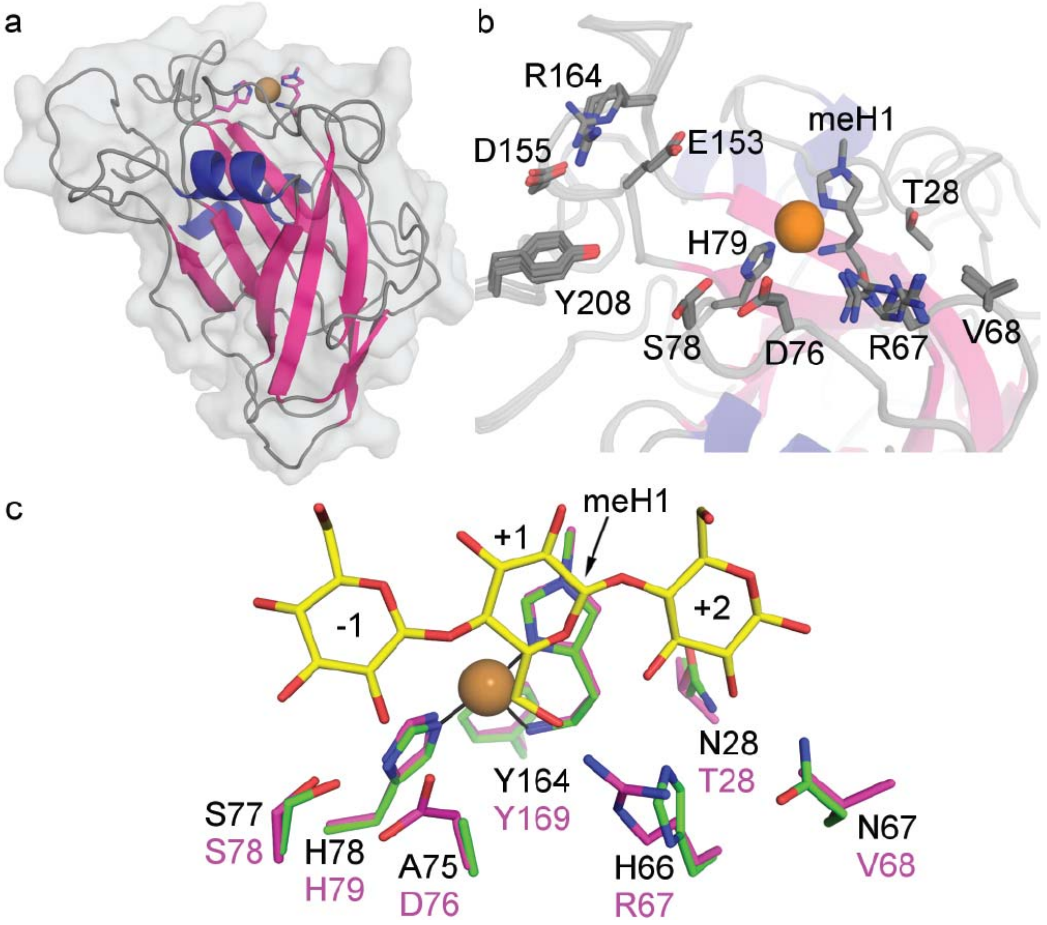

Supplementary Figure 10: *Cv*AA9A structure. a, Structure of *Cv*AA9A. **b**, *Cv*AA9A active site. **c**, Structural comparison of residues in *Ls*AA9A and *Cv*AA9A. Residues of *Ls*AA9A (green, black labels) interacting with $Cell_5$ (yellow) at subsite -1 to +2 and those equivalent in *Cv*AA9 (chain A - magenta) are shown (part of $Cell_5$ is not shown for clarity)..

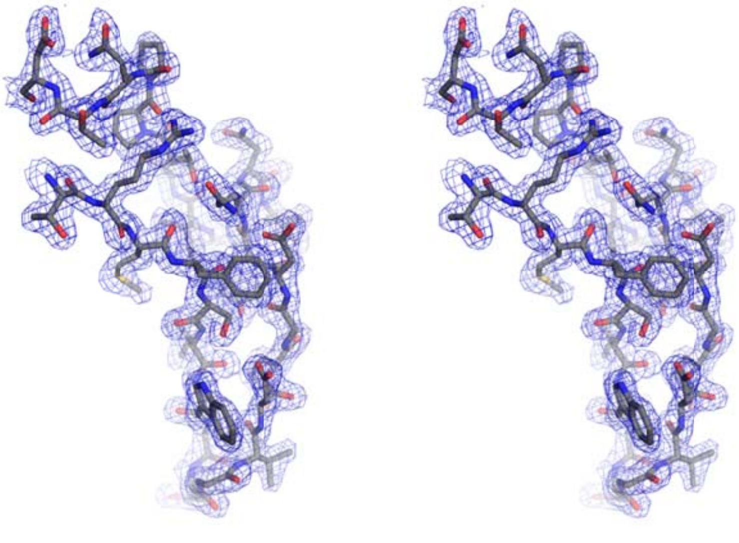

Supplementary Figure 11: Stereo view of representative electron density for the CvAA9A structure determined at 1.90 Å resolution. The region shown covers residues 2-29. The $2F_{obs}$-F_{calc} electron density map is shown contoured at 1σ. Difference electron density is also displayed at +3σ (green) and -3σ (red) but is barely visible.

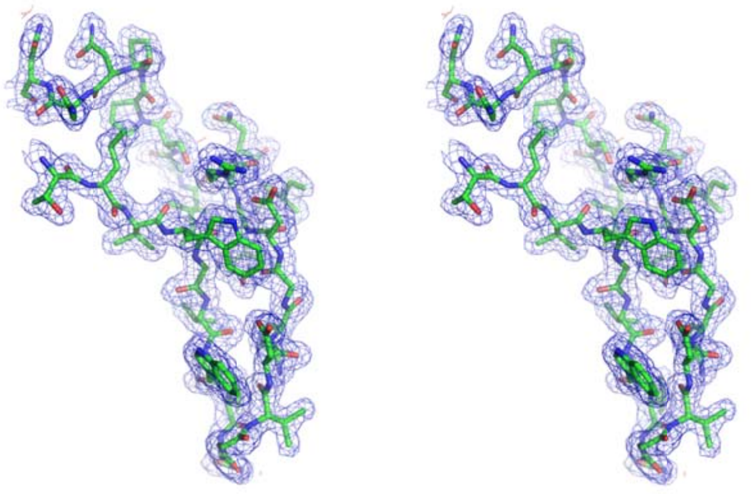

Supplementary Figure 12: Stereo view of representative electron density for the lowest resolution LsAA9A-oligosaccharide complex structure, LsAA9A:G4G4G3G determined at 2.0 Å resolution. The region shown covers residues 2-29. The $2F_{obs}$-F_{calc} electron density map is shown contoured at 1σ. Difference electron density is also displayed at +3σ (green) and -3σ (red) but is barely visible.

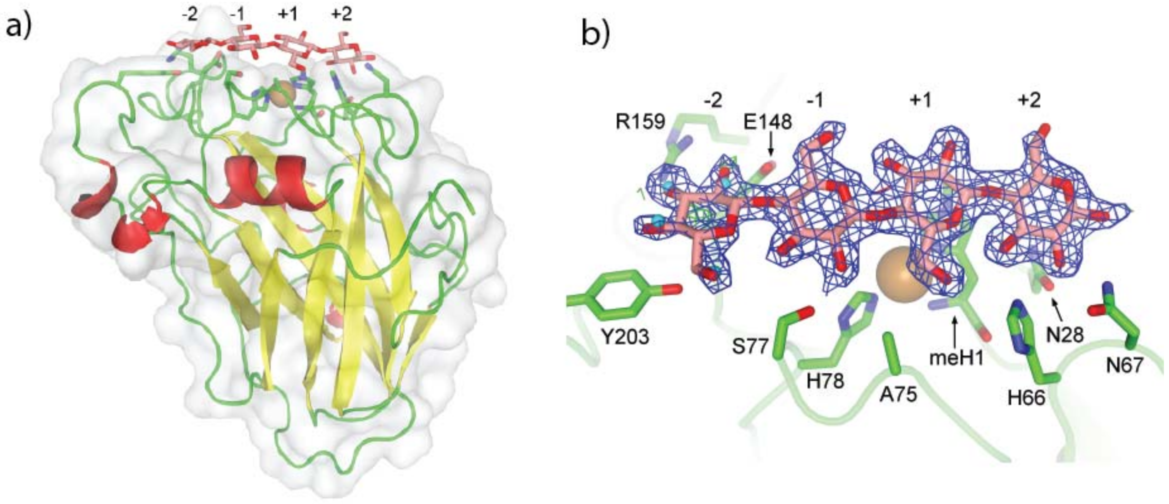

Supplementary Figure 13: Structure of LsAA9A:MLG4. a, Structure of *Ls*AA9A with MLG4 bound. **b,** zoom-in on active site of *Ls*AA9A with MLG4 bound. The $2F_{obs}$-F_{calc} electron density map contoured at 1σ shows β-(1→4)-glucan density, with no evidence of bound residues with β-(1→3)-linkages.

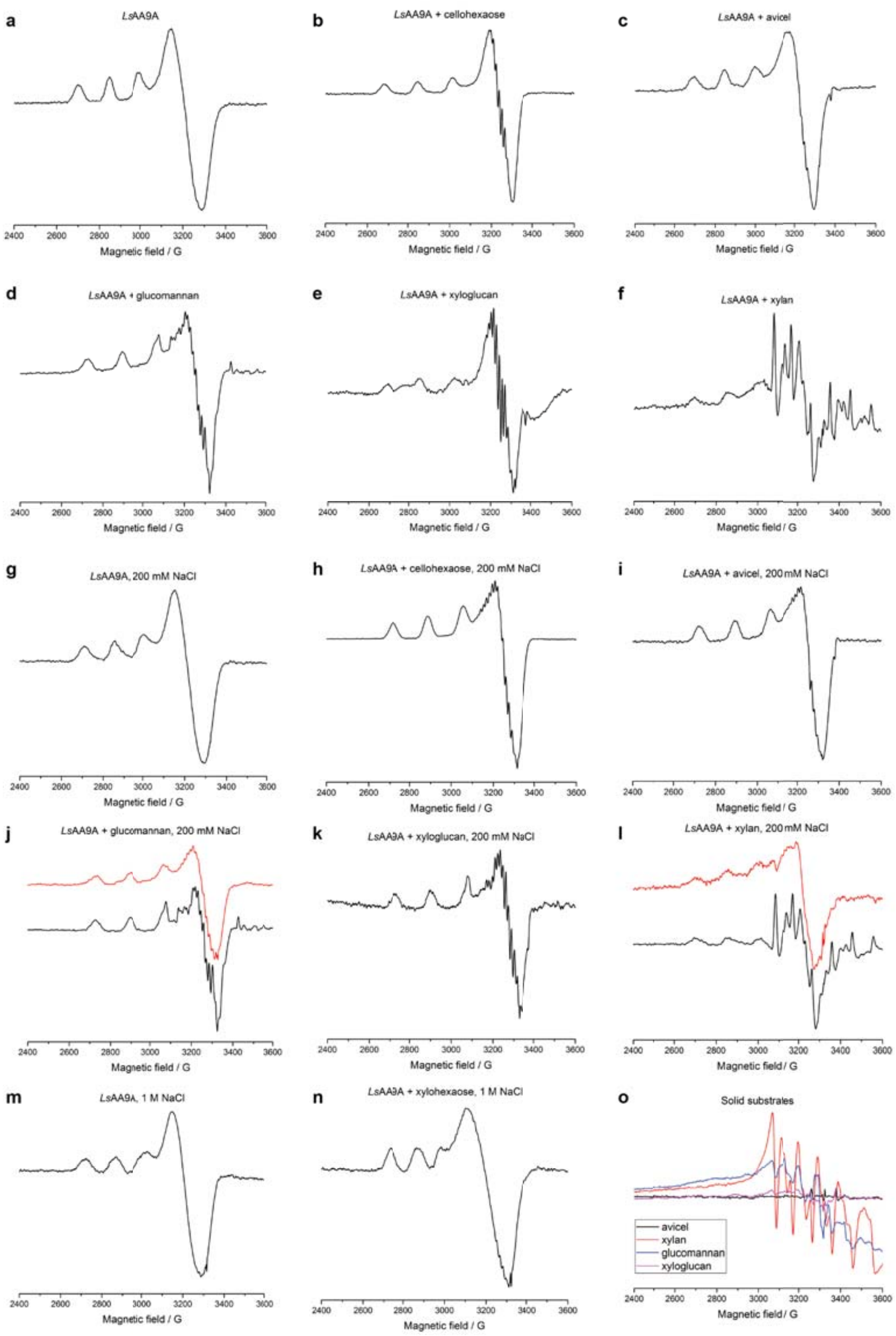

Supplementary Figure 14: X-band cw EPR spectra of *Ls*AA9A and various substrates. Spectra **a-f** were run in chloride-free buffer, spectra **g-l** in the presence of 200 mM NaCl, spectra **m** and **n** with 1 M NaCl. **a**. *Ls*AA9A. **b**. *Ls*AA9A in the presence of 2 equivalents of $Cell_6$. **c**. *Ls*AA9A with avicel. **d**. *Ls*AA9A and glucomannan. **e**. *Ls*AA9A and xyloglucan. **f**. *Ls*AA9A and xylan. **g**. *Ls*AA9A. **h**. *Ls*AA9A in the presence of 2 equivalents of $Cell_6$. **i**. *Ls*AA9A with avicel. **j**. *Ls*AA9A and glucomannan or solubilized glucomannan (red). **k**. *Ls*AA9A and xyloglucan. **l**. *Ls*AA9A and xylan or solubilized xylan (red). **m**. *Ls*AA9A. **n**. *Ls*AA9A in the presence of ~150 equivalents of Xyl_6. **o**. solid substrates.

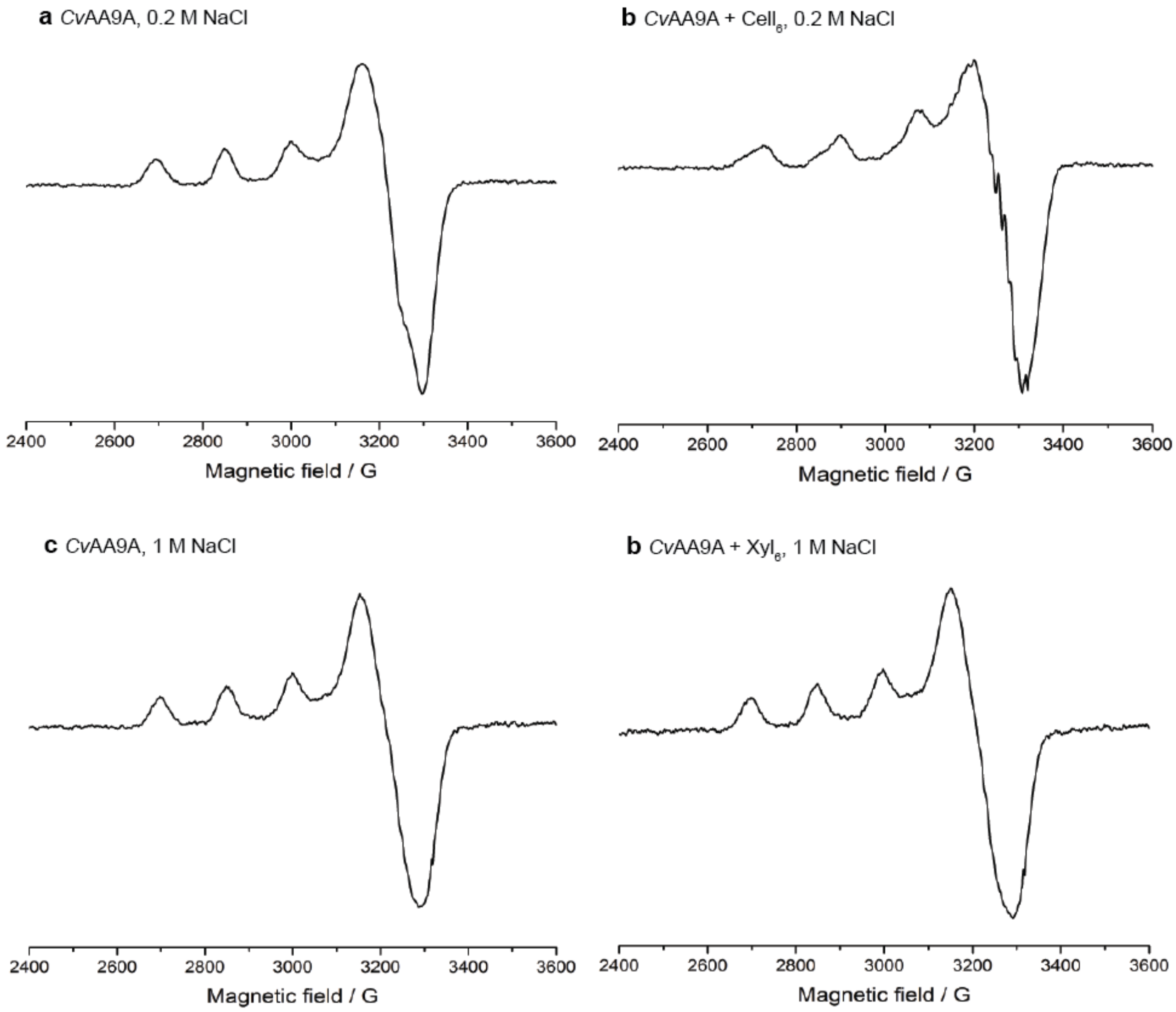

Supplementary Figure 15: X-band cw EPR spectra of *Cv*AA9A. Spectra **a** and **b** run in the presence of 200 mM NaCl, spectra **c** and **d** with 1 M NaCl. **a**. *Cv*AA9A. **b**. *Cv*AA9A in the presence of 4 equivalents of $Cell_6$. **c**. *Cv*AA9A. **d**. *Cv*AA9A in the presence of ~150 equivalents of Xyl_6

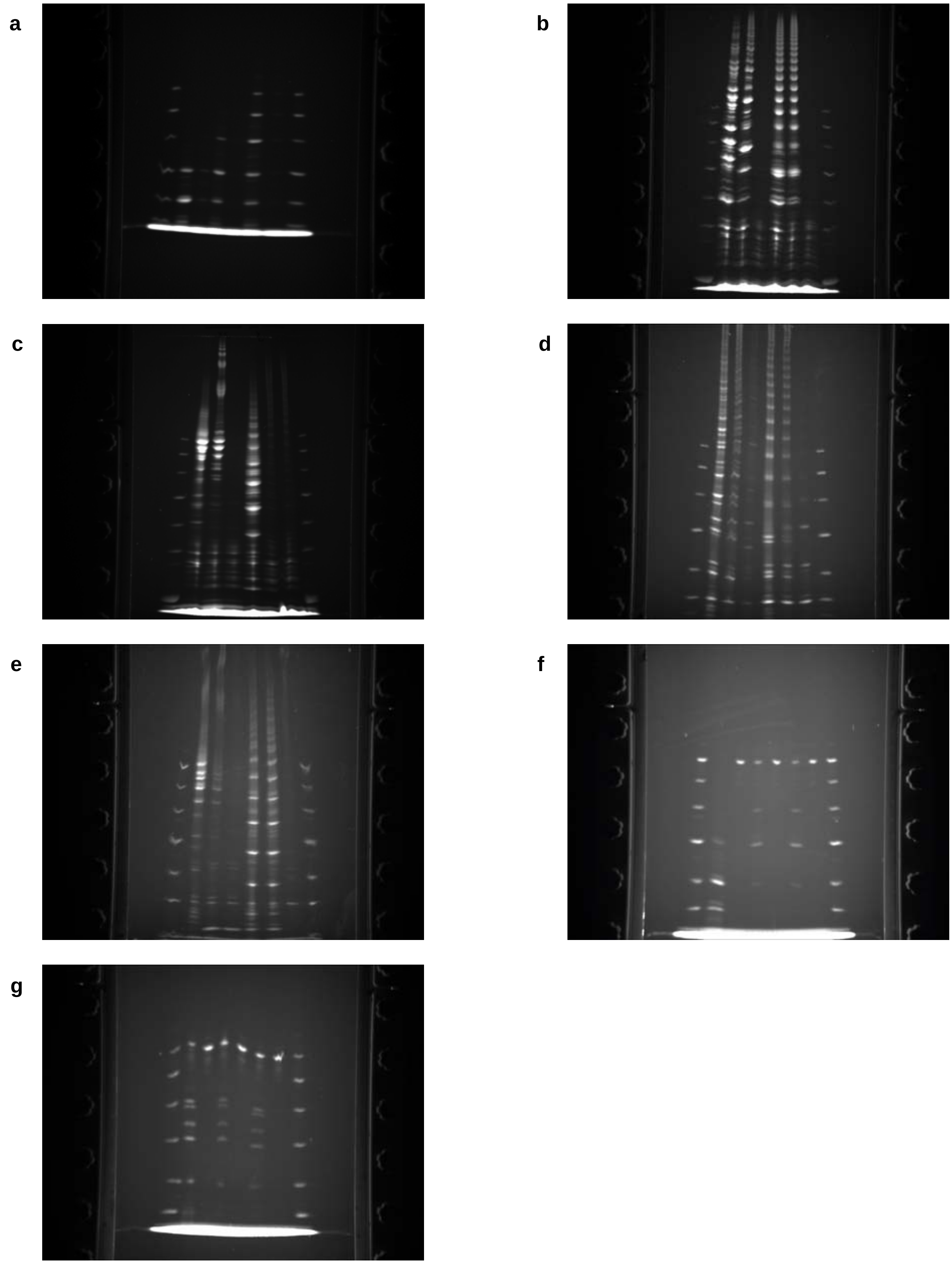

Supplementary Figure 16: Uncropped PACE gels. a. PACE gel from Fig. 1a. **b,c.** PACE gels from Fig. 2a. **d,e**. PACE gels from Fig. 4a. **f,g**. PACE gels from Fig. 4b.

Supplementary Table 1: Accession numbers of characterized LPMOs in the phylogenetic tree of Figure 1.

Label	Accession
**GH61-1**	EAA30263.1
**GH61-2**	EAA29018.1
**LsAA9A**	ALN96977.1
**MtLPMO9A**	AKO82493.1
**MYCTH_112089**	AEO60271.1
**MYCTH_92668**	AEO56665.1
**NcLPMO9C**	EAA36362.1
**NcLPMO9D**	CAD21296.1
**NcLPMO9E**	EAA26873.1
**NcLPMO9F**	CAD70347.1
**NcLPMO9M**	EAA33178.1
**NCU00836**	EAA34466.1
**PaLPMO9A**	CAP73254.1
**PaLPMO9E**	CAP67740.1
**PaLPMO9H**	CAP61476.1
**PaLPMO9D**	BAL43430.1
**PaLPMO9A**	ACS05720.1

Supplementary Table 2: glycosidic torsion angles

Glycosidic torsion angles		*Ls*AA9A:Cell$_5$	*Ls*AA9A:G4G4G 3G	*Ls*AA9A-Cu(II) :Xyl$_5$	*Ls*AA9A:Xyl$_5$	*Ls*AA9A:Xyl$_4$	*Ls*AA9A:Xyl$_3$	*Ls*AA9A:GM
Subsite +2/+3 (°)	Φ<br>Ψ	-<br>-	-<br>-	-<br>-	-<br>-	-<br>-	-<br>-	-83.5/-76.7 §<br>100.9/114.4 §
Subsite +1/+2 (°)	Φ<br>Ψ	-89.4<br>91.7	-92.6<br>93.3	-79.7<br>-175.6	-75.5<br>-175.4	-<br>-	-83.6<br>140.7	-93.7<br>94.9
Subsite -1/+1 (°)	Φ<br>Ψ	-83.9<br>99.9	-85.1<br>96.2	-75.1<br>152.5	-80.2<br>162.4	-<br>-	*(-103.6)**<br>*(164.7)**	-82.7<br>103.3
Subsite -2/-1 (°)	Φ<br>Ψ	-79.0<br>98.5	-66.9<br>94.2	-84.4<br>123.1	-86.3<br>118.7	-86.3<br>107.2	-87.2<br>99.4	-73.4<br>94.8
Subsite -3/-2 (°)	Φ<br>Ψ	-68.7<br>101.0	-<br>-	-98.1<br>127.0	-97.7<br>130.2	-113.3<br>132.3	-129.2<br>152.4	-113.7<br>126.7
Subsite -4/-3 (°)	Φ<br>Ψ	-<br>-	-<br>-	-<br>-	-<br>-	-<br>-	-<br>-	-92.6/-91.2 §<br>106.1/105.3 §
**Ideal cellulose torsion angles**	Φ<br>Ψ				-88.9<br>95.0			
**Definitions**	Φ<br>Ψ				$O_{5'} – C_{1'} – O_4 – C_4$<br>$C_{1'} – O_4 – C_4 – C_3$			
**Ideal xylan torsion angles**	Φ<br>Ψ				-<br>-			
**Definitions**	Φ<br>Ψ				$O_{5'} – C_{1'} – O_4 – C_4$<br>$C_{1'} – O_4 – C_4 – C_3$			

§ Alternative conformation
(xylosyl unit flipped out of subsite-1)

Supplementary Table 3: **Protein-substrate interactions**.

Potential hydrogen bonding distances (within 3.2 Å distance) in *Ls*AA9A-complex structures

Subsite	Glycosidic/	***Ls*AA9A:Cell$_5$** Residue (atom)	Distances (Å)	***Ls*AA9A:G4G4G3G** Residue (atom)	Distances (Å)	***Ls*AA9A-Cu(II):Xyl$_5$** Residue (atom)	Distances (Å)	***Ls*AA9A:Xyl$_5$** Residue (atom)	Distances (Å)	***Ls*AA9A:Xyl$_4$** Residue (atom)	Distances (Å)	***Ls*AA9A:Xyl$_3$** Residue (atom)	Distances (Å)	***Ls*AA9A:GM** Residue (atom)	Distances (Å)
**+3**	O(6)	-	-	-	-	n/a	n/a	n/a	n/a	n/a	n/a	n/a	n/a	H_2O (Asn28(N))	2.70§
	O(6)	-	-	-	-	n/a	n/a	n/a	n/a	n/a	n/a	n/a	n/a	Asn67(Nδ_2)	3.17
**+2**	O(1)	H_2O (Asn67(Nδ_2))	2.30	H_2O (Asn67(Nδ_1))	3.06	Asn28(Nδ_2)	2.94	Asn28(Nδ_2)	2.97	Asn28(Nδ_2)	3.06	Asn28(Nδ_2)	2.95	-	-
	O(1)	-	-	-	-	Asn67(Oδ_2)	2.67	Asn67(Nδ_2)	2.69	Asn67(Nδ_2)	2.71	Asn67(Nδ_2)	2.62	-	-
	O(2)	Asn28(Nδ_2)	2.81	Asn28(Nδ_2)	2.87	-	-	-	-	-	-	-	-	Asn28(Nδ_2)	2.89
	O(2)	Asn67(Nδ_2)	2.58	Asn67(Nδ_1)	2.57	-	-	-	-	-	-	-	-	Asn67(Nδ_2)	2.67
	O(3)	His66(Nε_2)	2.80	His66(Nε_2)	2.72	-	-	-	-	-	-	-	-	His66(Nε_2)	2.81
	O(4)	-	-	-	-	-	-	-	-	-	-	-	-	-	-
	O(4)	-	-	-	-	-	-	-	-	-	-	-	-	-	-
	O(5)	-	-	-	-	His66(Nε_2)	2.80	His66(Nε_2)	2.76	His66(Nε_2)	2.76	His66(Nε_2)	2.75	-	-
**+1**	O(6)	H_2O_{pocket}	2.80	H_2O_{pocket}	2.71	n/a	n/a	n/a	n/a	n/a	n/a	n/a	n/a	H_2O_{pocket}	2.83
**–1**	O(2)	Ser77(Oγ)	2.54	Ser77(Oγ)	2.59	Ser77(Oγ)	2.65	Ser77(Oγ)	2.63	Ser77(Oγ)	2.68	Ser77(Oγ)	2.57	Ser77(Oγ)	2.67
	O(2)	-	-	-	-	-	-	-	-	-	-	**(Gln162((Nδ_2))*	*(3.16)*	-	-
	O(3)	-	-	-	-	-	-	-	-	-	-	**(Glu148((Oε_2))*	*(2.60)*	-	-
	O(3)	-	-	-	-	H_2O (Tyr203(OH))	2.94	H_2O (Tyr203(OH))	2.95	-	-	**(Ile158((CO))*	*(3.04)*	-	-
	O(4)	-	-	-	-	-	-	-	-	-	-	**(Ile158((CO))*	*(2.87)*	-	-
	O(6)	-	-	H_2O (Glu148(Oε_1))	3.12	n/a	n/a	n/a	n/a	n/a	n/a	n/a	n/a	-	-
**–2**	O(2)	Glu148(Oε_1)	2.58	Glu148(Oε_1)	2.62	Glu148(Oε_1)	2.62	Glu148(Oε_1)	2.66	Glu148(Oε_1)	2.71	Glu148(Oε_1)	2.74	Glu148(Oε_1)	2.55
	O(2)	-	-	-	-	Arg159(Nω_1)	2.90	Arg159(Nω_1)	2.98	Arg159(Nω_1)	2.90	Arg159(Nω_1)	2.94	-	-
	O(3)	Arg159(Nω_2)	3.02	Arg159(Nω_2)	3.12	Arg159(Nω_2)	2.83	Arg159(Nω_2)	2.93	Arg159(Nω_2)	2.87	Arg159(Nω_2)	2.82	Arg159(Nω_1)	3.13
	O(3)	-	-	-	-	#Leu222(CO)	3.01	-	-	#Leu222(CO)	2.82	#Leu222(CO)	2.80	H_2O (Asp150(Oδ_2))	3.04
	O(5)	-	-	-	-	H_2O (Tyr203(OH))	2.80	H_2O (Tyr203(OH))	2.67	-	-	-	-	-	-
	O(6)	-	-	-	-	n/a	n/a	n/a	n/a	na	n/a	n/a	n/a	#Gly233(N)	2.71
**–3**	O(5)	-	-	-	-	-	-	H_2O (Asp150(Oδ_2))	2.86	-	-	-	-	-	-
	O(6)	Arg159(Nω_2)	3.01	-	-	n/a	n/a	-	-	n/a	n/a	n/a	n/a	-	-
	O(6)	Asp150(Oδ_2)	2.71	-	-	n/a	n/a	n/a	n/a	n/a	n/a	n/a	n/a	-	-
	O(6)	#Leu222(CO)	2.60	-	-	n/a	n/a	n/a	n/a	n/a	n/a	n/a	n/a	-	-
	O(2)	-	-	-	-	-	-	-	-	-	-	-	-	Asn201(Nδ_2)	3.13
	O(2)	-	-	-	-	-	-	-	-	-	-	-	-	Asn201(Nδ_2)	3.12 §
	O(3)	-	-	-	-	-	-	-	-	-	-	-	-	#Ser219(CO)	2.99
	O(3)	-	-	-	-	-	-	-	-	-	-	-	-	#Ser219(CO)	3.07 §

§ Alternative conformation; * *(xylosyl unit flipped out of subsite-1)*; *# Symmetry related interaction; n/a not applicable*

Supplementary Table 4: Crystallization and soaking conditions

**Crystallization Conditions**	***Ls*AA9A:Cell$_5$**	***Ls*AA9A:G4G4G3G**	***Ls*AA9A:Xyl$_3$**	***Ls*AA9A:Xyl$_4$**	***Ls*AA9A:Xyl$_5$**	***Ls*AA9A:Xyl$_5$ Cu(II)**	***Ls*AA9A:GM**	***Cv*AA9**
Protein concentration	19.2 mg/mL	19.2 mg/mL	19.2 mg/mL	19.2 mg/mL	19.2 mg/mL	19.2 mg/mL	19.2 mg/mL	6.3 mg/mL
Preincubation [Cu(II)acetate] Time	1.0 mM 1 hour	1.4 mM 30 min	1.0 mM 1 hour	1.0 mM 1 hour	1.0 mM 1 hour	1.0 mM 1 hour	1.0 mM 1 hour	1.0 mM 1 hour
Precipitant concentration	3.2 M NaCl	3.5 M NaCl	4.4 M NaCl	4.1 M NaCl	3.6 M NaCl	3.6 M NaCl	3.3 M NaCl	1.6 M $(NH_4)_2SO_4$ 0.1M NaCl
Reservoir buffer	0.1 M citric acid pH3.5	0.1 M citric acid pH3.5	0.1 M citric acid pH4.5	0.1 M citric acid pH4.0	0.1 M citric acid pH4.5	0.1 M citric acid pH4.5	0.1 M citric acid pH3.5	0.1 M HEPES pH 7.5
drop volume and ratio (Prot:Res:H_2O)	0.5 µL 3:1:1	0.4 µL 3:1:0	0.4 µL 3:1:0	0.5 µL 3:1:1	0.4 µL 3:1:0	0.4 µL 3:1:0	0.4 µL 3:1:0	0.5 µL 3:1:1
**Soaking Conditions**	***Ls*AA9A:Cell$_5$**	***Ls*AA9A:G4G4G3G**	***Ls*AA9A:Xyl$_3$**	***Ls*AA9A:Xyl$_4$**	***Ls*AA9A:Xyl$_5$**	***Ls*AA9A:Xyl$_5$ Cu(II)**	***Ls*AA9A:GM**	***Cv*AA9**
pH equilibration	30 min. in 0.4ul drop of pH 5.5	-	-	-	-	-	-	-
Soaking Conditions	1ul G5 solution added for 10 min	0,5ul of 0.3M MLG-B (reservoir) added for 20 min	1.45 M X3 pH5.5 1 hour	1.25 M X4 pH5.51 hour	0.9M X5 pH 5.5 10 min	0.9M X5 pH 5.5 10 min	GM solution pH5.5 15 min	-

Both proteins were buffered in 20 mM acetate pH 5.5

Supplementary Reference

1. Fry, S.C. *et al.* An unambiguous nomenclature for xyloglucan-derived oligosaccharides *Physiol. Plant.* **89**, (1993)
